# {*rac*-5-[Meth­oxy(phen­yl)meth­yl]-10,20-diphenyl­porphyrinato}nickel(II)

**DOI:** 10.1107/S1600536811002960

**Published:** 2011-01-29

**Authors:** Mathias O. Senge, Katja Dahms

**Affiliations:** a SFI Tetrapyrrole Laboratory, School of Chemistry, Trinity College Dublin, Dublin 2, Ireland

## Abstract

The title compound, [Ni(C_40_H_28_N_4_O)], was obtained from a Grignard reaction of the respective formyl­porphyrin to yield {5-[hy­droxy(phen­yl)meth­yl]-10,20-diphenyl­porphyrinato}nickel(II), followed by crystallization from methyl­ene chloride/methanol. The mol­ecule exhibits a ruffled macrocycle with an average deviation of the 24 macrocycle atoms from their least-squares plane (Δ24) of 0.26 Å and an average Ni—N bond length of 1.931 (2) Å. In line with the asymmetrical substituent pattern, the degree of distortion is slightly larger at point of attachment of the meth­oxy(phen­yl)methyl residue than at the unsubstituted *meso* position. The meth­oxy group attached to the chiral C atom is disordered in a 0.534 (4):0.466 (4) ratio.

## Related literature

For related literature on the conformation of porphyrins, see: Senge (2000 ▶). For the chemistry of porphyrins with mixed *meso* substituents, see: Dahms *et al.* (2007 ▶); Senge *et al.* (2010 ▶). For Ni(II) porphyrin structures, see: Fleischer *et al.* (1964 ▶); Gallucci *et al.* (1982 ▶); Hoard (1973 ▶); Lee & Scheidt (1987 ▶), Senge (2000 ▶) and Senge *et al.* (2000 ▶). For handling of the crystals, see: Hope (1994 ▶).
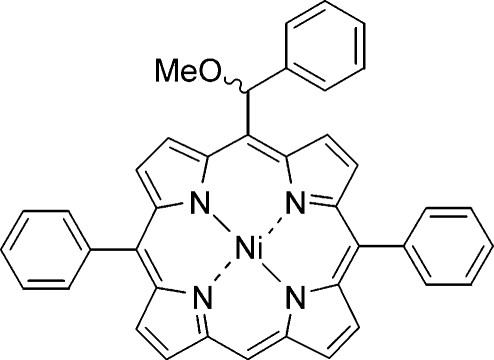


## Experimental

### 

#### Crystal data

[Ni(C_40_H_28_N_4_O)]
*M* *_r_* = 639.37
Triclinic, 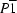

*a* = 10.869 (2) Å
*b* = 11.984 (2) Å
*c* = 12.332 (3) Å
α = 72.356 (6)°
β = 85.305 (8)°
γ = 74.219 (7)°
*V* = 1473.0 (5) Å^3^
*Z* = 2
Mo *K*α radiation
μ = 0.70 mm^−1^
*T* = 123 K
0.20 × 0.20 × 0.20 mm

#### Data collection

Rigaku Saturn724 diffractometer
Absorption correction: multi-scan (*CrystalClear*; Rigaku, 2007 ▶) *T*_min_ = 0.873, *T*_max_ = 0.873
29300 measured reflections
7270 independent reflections
6754 reflections with *I* > 2σ(*I*)
*R*_int_ = 0.040

#### Refinement

*R*[*F* ^2^ > 2σ(*F* ^2^)] = 0.053
*wR*(*F* ^2^) = 0.108
*S* = 1.10
7270 reflections
436 parameters
H-atom parameters constrained
Δρ_max_ = 0.37 e Å^−3^
Δρ_min_ = −0.52 e Å^−3^

### 

Data collection: *CrystalClear* (Rigaku, 2007 ▶); cell refinement: *CrystalClear*; data reduction: *CrystalClear*; program(s) used to solve structure: *SHELXS97* (Sheldrick, 2008 ▶); program(s) used to refine structure: *SHELXL97* (Sheldrick, 2008 ▶); molecular graphics: *SHELXTL* (Sheldrick, 2008 ▶); software used to prepare material for publication: *SHELXL97*.

## Supplementary Material

Crystal structure: contains datablocks I, global. DOI: 10.1107/S1600536811002960/go2001sup1.cif

Structure factors: contains datablocks I. DOI: 10.1107/S1600536811002960/go2001Isup2.hkl

Additional supplementary materials:  crystallographic information; 3D view; checkCIF report

## Figures and Tables

**Fig. 1.**
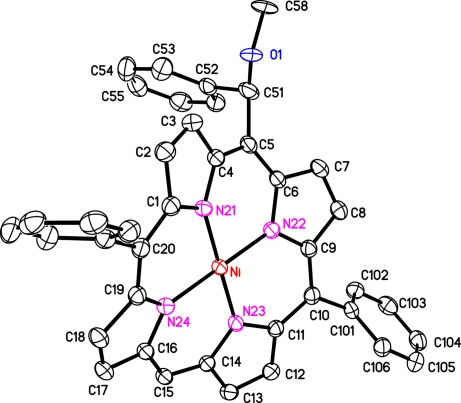
: View of the molecular structure of I in the crystals. Thermal ellipsoids are drawn for 50% occupancy. Only one of the two enantiomeric forms is shown; hydrogen atoms have been omitted for clarity.

**Fig. 2.**
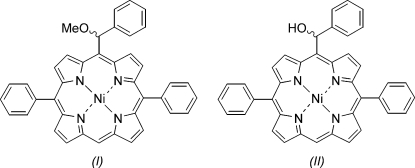
Schematic representations of (I) and (II).

## supplementary crystallographic information

### Comment

The title compound (I) crystallized as the racemic form in the triclinic space group *P*1. It was obtained from a Grignard reaction of the respective formylporphyrin to yield {5-[hydroxy(phenyl)methyl]-10,20-diphenylporphyrinato}nickel(II), (II) (Fig. 2), followed by crystallization from methylene chloride/methanol. *I.e.*, substitution of the hydroxy group by a methoxy group occurred during the crystallization. The stucture of the title compound, (I), is shown below. Dimensions are available in the archived CIF.

The molecule exhibits a ruffled macrocycle with an average deviation of the 24 macrocycle atoms from their least-squares-plane (Δ24) of 0.26 Å and an average Ni–N bond length of 1.931 (2) Å. In line with the unsymmetrical substituent pattern the degree of distortion is slightly larger at C5 (the methoxyphenylmethyl residue) then at C15 (the unsubstituted *meso* position). This is indicated by the individual displacements of the C_m_ positions from the least-squares-plane of the four nitrogen atoms. The respective displacement values are -0.64, 0.49, -0.49, 0.47 Å for C5, C10, C15 and C20, respectively. Similarly, the C_a_—C_m_—C_a_ angle for C15 is widened (123.2 (2)°) compared to the other three *meso* positions (average = 121.3 (2)°). In terms of macrocycle distortion modes, the most significant out-of-plane contributor is *B*_1u_ (ruffled) with some degree of *B*_2u_ (saddle) mixed in. The most prominent in-plane distortion mode is *A*_1 g_, *i.e.*, macrocycle breathing.

The molecules form a close spaced lattice structure characterized by stacking of the porphyrin macrocycles (not shown). The closest intramolecular contacts are Ni–H15 (3.034 Å) and Ni–H203 (2.764 Å). The former is a side-on contact and blocks one face of the porphyrin. The latter involves a *meta*-phenyl hydrogen atom pointing towards the nickel(II) center.

### Experimental

The title compound I was obtained from II (Dahms *et al.*, 2007) upon crystallization from CH_2_Cl_2_/CH_3_OH. Porphyrin II in turn was prepared *via* Grignard reaction of (5-formyl-10,20-diphenylporphyrinato)nickel(II) with phenyl magnesium bromide.

### Refinement

The compound crystallized with crystallographic disorder of the methoxy group at the *meso* carbon (C51) with the site-occupancy factors of 0.533 (3) and 0.467 (3) for part A and B respectively. The H atoms bonded to C58 and C58*a* atoms were refined with standard distances of 0.97 Å, for methyl groups with *U*_iso_(H)=1.5Ueq(C) and the H atom for C51 was refined with 0.98Å with *U*_iso_(H)=1.2 *U*_eq_(C).

### Crystal data

**Table:**

[Ni(C_40_H_28_N_4_O)]	*Z* = 2
*M**_r_* = 639.37	*F*(000) = 664
Triclinic, *P*1	*D*_x_ = 1.442 Mg m^−^^3^
Hall symbol: -P 1	Mo *K*α radiation, λ = 0.7107 Å
*a* = 10.869 (2) Å	Cell parameters from 4706 reflections
*b* = 11.984 (2) Å	θ = 2.0–28.3°
*c* = 12.332 (3) Å	µ = 0.70 mm^−^^1^
α = 72.356 (6)°	*T* = 123 K
β = 85.305 (8)°	Prism, red
γ = 74.219 (7)°	0.20 × 0.20 × 0.20 mm
*V* = 1473.0 (5) Å^3^	

### Data collection

**Table:**

Rigaku Saturn724 diffractometer	7270 independent reflections
Radiation source: Sealed Tube	6754 reflections with *I* > 2σ(*I*)
Graphite Monochromator	*R*_int_ = 0.040
Detector resolution: 28.5714 pixels mm^-1^	θ_max_ = 28.4°, θ_min_ = 2.6°
dtprofit.ref scans	*h* = −14→14
Absorption correction: multi-scan (*CrystalClear*; Rigaku, 2007)	*k* = −16→15
*T*_min_ = 0.873, *T*_max_ = 0.873	*l* = −16→16
29300 measured reflections	

### Refinement

**Table:**

Refinement on *F*^2^	Primary atom site location: structure-invariant direct methods
Least-squares matrix: full	Secondary atom site location: difference Fourier map
*R*[*F*^2^ > 2σ(*F*^2^)] = 0.053	Hydrogen site location: inferred from neighbouring sites
*wR*(*F*^2^) = 0.108	H-atom parameters constrained
*S* = 1.10	*w* = 1/[σ^2^(*F*_o_^2^) + (0.0324*P*)^2^ + 1.2452*P*] where *P* = (*F*_o_^2^ + 2*F*_c_^2^)/3
7270 reflections	(Δ/σ)_max_ = 0.001
436 parameters	Δρ_max_ = 0.37 e Å^−^^3^
0 restraints	Δρ_min_ = −0.52 e Å^−^^3^

### Special details

**Table:**

Geometry. All e.s.d.'s (except the e.s.d. in the dihedral angle between two l.s. planes) are estimated using the full covariance matrix. The cell e.s.d.'s are taken into account individually in the estimation of e.s.d.'s in distances, angles and torsion angles; correlations between e.s.d.'s in cell parameters are only used when they are defined by crystal symmetry. An approximate (isotropic) treatment of cell e.s.d.'s is used for estimating e.s.d.'s involving l.s. planes.
Refinement. Refinement of *F*^2^ against ALL reflections. The weighted *R*-factor *wR* and goodness of fit *S* are based on *F*^2^, conventional *R*-factors *R* are based on *F*, with *F* set to zero for negative *F*^2^. The threshold expression of *F*^2^ > σ(*F*^2^) is used only for calculating *R*-factors(gt) *etc*. and is not relevant to the choice of reflections for refinement. *R*-factors based on *F*^2^ are statistically about twice as large as those based on *F*, and *R*- factors based on ALL data will be even larger. The compound crystallized with crystallographic disorder of the methoxy group at the *meso* carbon (C51) with the site-occupancy factors of 0.533 (3) and 0.467 (3) for part A and B respectively. The H atoms bonded to C58 and C58*a* atoms were refined with standard distances of 0.97 Å, for methyl groups with *U*_iso_(H)=1.5Ueq(C) and the H atom for C51 was refined with 0.98Å with *U*_iso_(H)=1.2 *U*_eq_(C).

### Fractional atomic coordinates and isotropic or equivalent isotropic displacement parameters (Å^2^)

**Table:**

	*x*	*y*	*z*	*U*_iso_*/*U*_eq_	Occ. (<1)
Ni	0.06269 (3)	0.66980 (3)	0.41138 (2)	0.02087 (8)	
O1	−0.1607 (3)	0.9874 (3)	−0.0116 (3)	0.0299 (8)	0.534 (4)
C58	−0.1951 (6)	1.0236 (5)	−0.1294 (4)	0.0373 (13)	0.534 (4)
H58A	−0.2379	1.1105	−0.1538	0.056*	0.534 (4)
H58B	−0.2531	0.9777	−0.1397	0.056*	0.534 (4)
H58C	−0.1179	1.0074	−0.1750	0.056*	0.534 (4)
O1A	−0.0393 (3)	0.8932 (3)	−0.0643 (3)	0.0282 (9)	0.466 (4)
C58A	−0.1124 (5)	0.9638 (5)	−0.1660 (4)	0.0317 (13)	0.466 (4)
H58D	−0.0571	0.9623	−0.2328	0.047*	0.466 (4)
H58E	−0.1464	1.0477	−0.1639	0.047*	0.466 (4)
H58F	−0.1833	0.9295	−0.1706	0.047*	0.466 (4)
N21	−0.06152 (17)	0.82009 (17)	0.34561 (15)	0.0224 (4)	
N22	0.13478 (17)	0.67312 (17)	0.26209 (15)	0.0224 (4)	
N23	0.18836 (17)	0.51926 (17)	0.47673 (15)	0.0217 (4)	
N24	−0.00879 (17)	0.66697 (17)	0.56101 (15)	0.0229 (4)	
C1	−0.1395 (2)	0.8957 (2)	0.40303 (19)	0.0249 (4)	
C2	−0.2243 (2)	0.9954 (2)	0.3252 (2)	0.0292 (5)	
H2	−0.2845	1.0612	0.3434	0.035*	
C3	−0.2025 (2)	0.9785 (2)	0.2211 (2)	0.0292 (5)	
H3	−0.2467	1.0287	0.1529	0.035*	
C4	−0.0998 (2)	0.8703 (2)	0.23274 (18)	0.0239 (4)	
C5	−0.0403 (2)	0.8278 (2)	0.14332 (18)	0.0240 (4)	
C6	0.0776 (2)	0.7423 (2)	0.15786 (18)	0.0229 (4)	
C7	0.1641 (2)	0.7195 (2)	0.06717 (19)	0.0275 (5)	
H7	0.1475	0.7538	−0.0121	0.033*	
C8	0.2729 (2)	0.6402 (2)	0.11593 (19)	0.0282 (5)	
H8	0.3486	0.6116	0.0774	0.034*	
C9	0.2530 (2)	0.6072 (2)	0.23683 (19)	0.0241 (4)	
C10	0.3340 (2)	0.5109 (2)	0.31438 (19)	0.0238 (4)	
C11	0.2962 (2)	0.4657 (2)	0.42545 (18)	0.0232 (4)	
C12	0.3591 (2)	0.3490 (2)	0.50041 (19)	0.0266 (5)	
H12	0.4356	0.2948	0.4854	0.032*	
C13	0.2880 (2)	0.3315 (2)	0.59614 (19)	0.0265 (5)	
H13	0.3039	0.2616	0.6606	0.032*	
C14	0.1843 (2)	0.4377 (2)	0.58233 (18)	0.0228 (4)	
C15	0.0995 (2)	0.4599 (2)	0.66710 (19)	0.0244 (4)	
H15	0.1007	0.3960	0.7354	0.029*	
C16	0.0134 (2)	0.5698 (2)	0.65786 (18)	0.0240 (4)	
C17	−0.0566 (2)	0.6025 (2)	0.7521 (2)	0.0295 (5)	
H17	−0.0589	0.5505	0.8272	0.035*	
C18	−0.1184 (2)	0.7206 (2)	0.7145 (2)	0.0312 (5)	
H18	−0.1695	0.7687	0.7586	0.037*	
C19	−0.0921 (2)	0.7607 (2)	0.59404 (19)	0.0248 (4)	
C20	−0.1506 (2)	0.8725 (2)	0.52061 (19)	0.0256 (5)	
C51	−0.1098 (2)	0.8703 (2)	0.0298 (2)	0.0336 (6)	
H51	−0.0424	0.8493	−0.0262	0.040*	0.534 (4)
H51A	−0.1685	0.9511	0.0283	0.040*	0.466 (4)
C52	−0.1998 (2)	0.7901 (2)	0.03594 (19)	0.0280 (5)	
C53	−0.3275 (2)	0.8242 (3)	0.0657 (2)	0.0375 (6)	
H53	−0.3621	0.9014	0.0775	0.045*	
C54	−0.4051 (3)	0.7458 (3)	0.0782 (2)	0.0425 (7)	
H54	−0.4926	0.7699	0.0980	0.051*	
C55	−0.3551 (3)	0.6327 (3)	0.0621 (2)	0.0386 (6)	
H55	−0.4075	0.5785	0.0723	0.046*	
C56	−0.2285 (3)	0.5993 (3)	0.0310 (2)	0.0380 (6)	
H56	−0.1941	0.5224	0.0184	0.046*	
C57	−0.1515 (2)	0.6774 (2)	0.0180 (2)	0.0320 (5)	
H57	−0.0646	0.6536	−0.0034	0.038*	
C101	0.4587 (2)	0.4433 (2)	0.27784 (18)	0.0240 (4)	
C102	0.4646 (2)	0.3764 (2)	0.2015 (2)	0.0281 (5)	
H102	0.3881	0.3775	0.1685	0.034*	
C103	0.5811 (2)	0.3085 (2)	0.1734 (2)	0.0321 (5)	
H103	0.5841	0.2637	0.1211	0.039*	
C104	0.6934 (2)	0.3057 (2)	0.2216 (2)	0.0328 (5)	
H104	0.7732	0.2597	0.2018	0.039*	
C105	0.6885 (2)	0.3702 (2)	0.2984 (2)	0.0323 (5)	
H105	0.7651	0.3674	0.3324	0.039*	
C106	0.5727 (2)	0.4388 (2)	0.3261 (2)	0.0288 (5)	
H106	0.5705	0.4834	0.3785	0.035*	
C201	−0.2389 (2)	0.9668 (2)	0.56703 (19)	0.0266 (5)	
C202	−0.2094 (2)	1.0755 (2)	0.5560 (2)	0.0307 (5)	
H202	−0.1335	1.0907	0.5176	0.037*	
C203	−0.2902 (2)	1.1623 (2)	0.6010 (2)	0.0344 (6)	
H203	−0.2697	1.2366	0.5927	0.041*	
C204	−0.4003 (2)	1.1408 (3)	0.6577 (2)	0.0373 (6)	
H204	−0.4542	1.1993	0.6901	0.045*	
C205	−0.4319 (2)	1.0338 (3)	0.6670 (2)	0.0391 (6)	
H205	−0.5083	1.0195	0.7048	0.047*	
C206	−0.3522 (2)	0.9476 (2)	0.6214 (2)	0.0346 (6)	
H206	−0.3750	0.8749	0.6271	0.041*	

### Atomic displacement parameters (Å^2^)

**Table:**

	*U*^11^	*U*^22^	*U*^33^	*U*^12^	*U*^13^	*U*^23^
Ni	0.02108 (14)	0.02495 (16)	0.01675 (14)	−0.00704 (11)	0.00045 (10)	−0.00553 (11)
O1	0.0398 (18)	0.0252 (16)	0.0224 (16)	−0.0079 (14)	−0.0088 (13)	−0.0017 (13)
C58	0.057 (3)	0.030 (3)	0.023 (2)	−0.014 (2)	−0.018 (2)	0.002 (2)
O1A	0.0301 (18)	0.037 (2)	0.0164 (16)	−0.0129 (16)	−0.0029 (13)	−0.0010 (15)
C58A	0.040 (3)	0.037 (3)	0.017 (2)	−0.017 (3)	−0.010 (2)	0.001 (2)
N21	0.0231 (9)	0.0254 (10)	0.0191 (9)	−0.0073 (7)	−0.0012 (7)	−0.0057 (8)
N22	0.0234 (9)	0.0257 (10)	0.0190 (9)	−0.0084 (7)	−0.0007 (7)	−0.0057 (8)
N23	0.0218 (8)	0.0264 (10)	0.0174 (8)	−0.0077 (7)	0.0009 (7)	−0.0060 (7)
N24	0.0232 (9)	0.0257 (10)	0.0188 (9)	−0.0064 (7)	0.0001 (7)	−0.0052 (8)
C1	0.0235 (10)	0.0269 (11)	0.0242 (11)	−0.0070 (9)	−0.0003 (8)	−0.0071 (9)
C2	0.0264 (11)	0.0285 (12)	0.0287 (12)	−0.0031 (9)	−0.0002 (9)	−0.0062 (10)
C3	0.0268 (11)	0.0302 (12)	0.0254 (11)	−0.0038 (10)	−0.0036 (9)	−0.0031 (10)
C4	0.0241 (10)	0.0270 (11)	0.0195 (10)	−0.0082 (9)	−0.0018 (8)	−0.0036 (9)
C5	0.0273 (11)	0.0255 (11)	0.0196 (10)	−0.0125 (9)	0.0013 (8)	−0.0028 (9)
C6	0.0271 (10)	0.0264 (11)	0.0169 (10)	−0.0117 (9)	0.0022 (8)	−0.0049 (9)
C7	0.0324 (12)	0.0297 (12)	0.0193 (10)	−0.0090 (10)	0.0024 (9)	−0.0051 (9)
C8	0.0309 (12)	0.0319 (13)	0.0218 (11)	−0.0089 (10)	0.0049 (9)	−0.0084 (10)
C9	0.0250 (10)	0.0275 (11)	0.0211 (10)	−0.0097 (9)	0.0023 (8)	−0.0070 (9)
C10	0.0238 (10)	0.0281 (12)	0.0222 (11)	−0.0096 (9)	0.0013 (8)	−0.0091 (9)
C11	0.0219 (10)	0.0275 (11)	0.0213 (10)	−0.0073 (9)	0.0004 (8)	−0.0080 (9)
C12	0.0244 (10)	0.0282 (12)	0.0257 (11)	−0.0056 (9)	−0.0005 (9)	−0.0069 (10)
C13	0.0269 (11)	0.0279 (12)	0.0230 (11)	−0.0080 (9)	−0.0018 (9)	−0.0036 (9)
C14	0.0229 (10)	0.0259 (11)	0.0193 (10)	−0.0083 (9)	−0.0014 (8)	−0.0040 (9)
C15	0.0243 (10)	0.0292 (12)	0.0191 (10)	−0.0103 (9)	0.0006 (8)	−0.0036 (9)
C16	0.0248 (10)	0.0287 (12)	0.0179 (10)	−0.0099 (9)	0.0007 (8)	−0.0035 (9)
C17	0.0305 (11)	0.0362 (13)	0.0197 (11)	−0.0074 (10)	0.0033 (9)	−0.0068 (10)
C18	0.0333 (12)	0.0366 (13)	0.0202 (11)	−0.0041 (10)	0.0029 (9)	−0.0085 (10)
C19	0.0243 (10)	0.0290 (12)	0.0217 (11)	−0.0064 (9)	0.0019 (8)	−0.0091 (9)
C20	0.0232 (10)	0.0288 (12)	0.0249 (11)	−0.0072 (9)	0.0009 (9)	−0.0082 (10)
C51	0.0384 (13)	0.0414 (15)	0.0207 (11)	−0.0202 (12)	−0.0057 (10)	0.0014 (11)
C52	0.0312 (12)	0.0362 (13)	0.0175 (10)	−0.0137 (10)	−0.0020 (9)	−0.0039 (10)
C53	0.0349 (13)	0.0457 (16)	0.0378 (14)	−0.0124 (12)	0.0019 (11)	−0.0196 (12)
C54	0.0331 (13)	0.0645 (19)	0.0391 (15)	−0.0219 (13)	0.0060 (11)	−0.0218 (14)
C55	0.0464 (15)	0.0505 (17)	0.0279 (13)	−0.0288 (13)	0.0011 (11)	−0.0104 (12)
C56	0.0460 (15)	0.0415 (15)	0.0306 (13)	−0.0152 (12)	−0.0032 (11)	−0.0124 (12)
C57	0.0314 (12)	0.0405 (14)	0.0249 (12)	−0.0099 (11)	−0.0016 (9)	−0.0099 (11)
C101	0.0243 (10)	0.0265 (11)	0.0204 (10)	−0.0082 (9)	0.0034 (8)	−0.0051 (9)
C102	0.0293 (11)	0.0308 (12)	0.0254 (11)	−0.0081 (10)	−0.0004 (9)	−0.0096 (10)
C103	0.0390 (13)	0.0303 (13)	0.0266 (12)	−0.0060 (11)	0.0036 (10)	−0.0112 (10)
C104	0.0299 (12)	0.0299 (13)	0.0329 (13)	−0.0039 (10)	0.0078 (10)	−0.0064 (11)
C105	0.0250 (11)	0.0379 (14)	0.0345 (13)	−0.0103 (10)	0.0018 (10)	−0.0100 (11)
C106	0.0274 (11)	0.0334 (13)	0.0288 (12)	−0.0112 (10)	0.0028 (9)	−0.0115 (10)
C201	0.0254 (11)	0.0326 (12)	0.0218 (11)	−0.0049 (9)	−0.0013 (9)	−0.0099 (10)
C202	0.0280 (11)	0.0344 (13)	0.0306 (12)	−0.0071 (10)	−0.0013 (10)	−0.0113 (11)
C203	0.0340 (13)	0.0359 (14)	0.0337 (13)	−0.0036 (11)	−0.0062 (10)	−0.0144 (11)
C204	0.0304 (12)	0.0461 (16)	0.0336 (13)	0.0036 (11)	−0.0042 (10)	−0.0200 (12)
C205	0.0243 (11)	0.0518 (17)	0.0403 (15)	−0.0053 (11)	0.0039 (11)	−0.0174 (13)
C206	0.0276 (12)	0.0398 (14)	0.0383 (14)	−0.0093 (11)	0.0025 (10)	−0.0144 (12)

### Geometric parameters (Å, °)

**Table:**

Ni—N21	1.9224 (19)	C15—C16	1.371 (3)
Ni—N23	1.9308 (19)	C15—H15	0.9500
Ni—N22	1.9343 (18)	C16—C17	1.432 (3)
Ni—N24	1.9368 (18)	C17—C18	1.344 (3)
O1—C51	1.314 (4)	C17—H17	0.9500
O1—C58	1.434 (5)	C18—C19	1.446 (3)
O1—H51A	0.5664	C18—H18	0.9500
C58—H58A	0.9800	C19—C20	1.384 (3)
C58—H58B	0.9800	C20—C201	1.496 (3)
C58—H58C	0.9800	C51—C52	1.529 (3)
O1A—C51	1.336 (4)	C51—H51	1.0000
O1A—C58A	1.440 (6)	C51—H51A	1.0000
C58A—H58D	0.9800	C52—C57	1.387 (3)
C58A—H58E	0.9800	C52—C53	1.388 (3)
C58A—H58F	0.9800	C53—C54	1.394 (4)
N21—C1	1.383 (3)	C53—H53	0.9500
N21—C4	1.387 (3)	C54—C55	1.384 (4)
N22—C9	1.380 (3)	C54—H54	0.9500
N22—C6	1.390 (3)	C55—C56	1.381 (4)
N23—C14	1.377 (3)	C55—H55	0.9500
N23—C11	1.379 (3)	C56—C57	1.384 (4)
N24—C16	1.376 (3)	C56—H56	0.9500
N24—C19	1.382 (3)	C57—H57	0.9500
C1—C20	1.393 (3)	C101—C102	1.398 (3)
C1—C2	1.432 (3)	C101—C106	1.398 (3)
C2—C3	1.352 (3)	C102—C103	1.387 (3)
C2—H2	0.9500	C102—H102	0.9500
C3—C4	1.440 (3)	C103—C104	1.389 (4)
C3—H3	0.9500	C103—H103	0.9500
C4—C5	1.392 (3)	C104—C105	1.383 (3)
C5—C6	1.391 (3)	C104—H104	0.9500
C5—C51	1.524 (3)	C105—C106	1.384 (3)
C6—C7	1.443 (3)	C105—H105	0.9500
C7—C8	1.350 (3)	C106—H106	0.9500
C7—H7	0.9500	C201—C202	1.390 (3)
C8—C9	1.436 (3)	C201—C206	1.394 (3)
C8—H8	0.9500	C202—C203	1.391 (3)
C9—C10	1.392 (3)	C202—H202	0.9500
C10—C11	1.385 (3)	C203—C204	1.381 (4)
C10—C101	1.490 (3)	C203—H203	0.9500
C11—C12	1.441 (3)	C204—C205	1.386 (4)
C12—C13	1.350 (3)	C204—H204	0.9500
C12—H12	0.9500	C205—C206	1.385 (3)
C13—C14	1.428 (3)	C205—H205	0.9500
C13—H13	0.9500	C206—H206	0.9500
C14—C15	1.375 (3)		
			
N21—Ni—N23	179.60 (8)	C16—C17—H17	126.4
N21—Ni—N22	89.80 (8)	C17—C18—C19	106.9 (2)
N23—Ni—N22	89.88 (8)	C17—C18—H18	126.6
N21—Ni—N24	90.21 (8)	C19—C18—H18	126.6
N23—Ni—N24	90.10 (8)	N24—C19—C20	124.8 (2)
N22—Ni—N24	179.66 (8)	N24—C19—C18	110.0 (2)
C51—O1—C58	113.3 (3)	C20—C19—C18	125.0 (2)
C51—O1—H51A	45.3	C19—C20—C1	121.4 (2)
C58—O1—H51A	136.1	C19—C20—C201	119.7 (2)
C51—O1A—C58A	114.3 (4)	C1—C20—C201	118.6 (2)
O1A—C58A—H58D	109.5	O1—C51—O1A	80.5 (2)
O1A—C58A—H58E	109.5	O1—C51—C5	116.8 (2)
H58D—C58A—H58E	109.5	O1A—C51—C5	117.0 (2)
O1A—C58A—H58F	109.5	O1—C51—C52	115.3 (2)
H58D—C58A—H58F	109.5	O1A—C51—C52	117.1 (2)
H58E—C58A—H58F	109.5	C5—C51—C52	108.33 (19)
C1—N21—C4	105.40 (18)	O1—C51—H51	105.1
C1—N21—Ni	126.87 (15)	C5—C51—H51	105.1
C4—N21—Ni	127.52 (15)	C52—C51—H51	105.1
C9—N22—C6	105.75 (17)	O1A—C51—H51A	104.2
C9—N22—Ni	127.32 (15)	C5—C51—H51A	104.2
C6—N22—Ni	126.94 (15)	C52—C51—H51A	104.2
C14—N23—C11	104.96 (18)	H51—C51—H51A	128.8
C14—N23—Ni	127.10 (14)	C57—C52—C53	118.9 (2)
C11—N23—Ni	127.79 (15)	C57—C52—C51	119.5 (2)
C16—N24—C19	105.15 (18)	C53—C52—C51	121.5 (2)
C16—N24—Ni	126.83 (15)	C52—C53—C54	120.3 (3)
C19—N24—Ni	128.03 (15)	C52—C53—H53	119.9
N21—C1—C20	126.4 (2)	C54—C53—H53	119.9
N21—C1—C2	110.34 (19)	C55—C54—C53	120.2 (3)
C20—C1—C2	122.6 (2)	C55—C54—H54	119.9
C3—C2—C1	107.1 (2)	C53—C54—H54	119.9
C3—C2—H2	126.4	C56—C55—C54	119.6 (3)
C1—C2—H2	126.4	C56—C55—H55	120.2
C2—C3—C4	107.3 (2)	C54—C55—H55	120.2
C2—C3—H3	126.3	C55—C56—C57	120.2 (3)
C4—C3—H3	126.3	C55—C56—H56	119.9
N21—C4—C5	124.7 (2)	C57—C56—H56	119.9
N21—C4—C3	109.72 (19)	C56—C57—C52	120.8 (2)
C5—C4—C3	125.4 (2)	C56—C57—H57	119.6
C6—C5—C4	121.2 (2)	C52—C57—H57	119.6
C6—C5—C51	119.7 (2)	C102—C101—C106	118.5 (2)
C4—C5—C51	119.0 (2)	C102—C101—C10	121.4 (2)
N22—C6—C5	125.21 (19)	C106—C101—C10	120.0 (2)
N22—C6—C7	109.45 (19)	C103—C102—C101	120.5 (2)
C5—C6—C7	125.1 (2)	C103—C102—H102	119.7
C8—C7—C6	107.3 (2)	C101—C102—H102	119.7
C8—C7—H7	126.4	C102—C103—C104	120.3 (2)
C6—C7—H7	126.4	C102—C103—H103	119.9
C7—C8—C9	107.3 (2)	C104—C103—H103	119.9
C7—C8—H8	126.3	C105—C104—C103	119.7 (2)
C9—C8—H8	126.3	C105—C104—H104	120.2
N22—C9—C10	125.3 (2)	C103—C104—H104	120.2
N22—C9—C8	110.0 (2)	C104—C105—C106	120.3 (2)
C10—C9—C8	124.1 (2)	C104—C105—H105	119.8
C11—C10—C9	121.4 (2)	C106—C105—H105	119.8
C11—C10—C101	117.1 (2)	C105—C106—C101	120.7 (2)
C9—C10—C101	121.2 (2)	C105—C106—H106	119.6
N23—C11—C10	125.6 (2)	C101—C106—H106	119.6
N23—C11—C12	110.31 (19)	C202—C201—C206	118.9 (2)
C10—C11—C12	123.9 (2)	C202—C201—C20	120.3 (2)
C13—C12—C11	106.7 (2)	C206—C201—C20	120.8 (2)
C13—C12—H12	126.6	C203—C202—C201	120.4 (2)
C11—C12—H12	126.6	C203—C202—H202	119.8
C12—C13—C14	107.1 (2)	C201—C202—H202	119.8
C12—C13—H13	126.4	C204—C203—C202	120.2 (2)
C14—C13—H13	126.4	C204—C203—H203	119.9
C15—C14—N23	124.6 (2)	C202—C203—H203	119.9
C15—C14—C13	124.2 (2)	C203—C204—C205	119.8 (2)
N23—C14—C13	110.83 (19)	C203—C204—H204	120.1
C16—C15—C14	123.2 (2)	C205—C204—H204	120.1
C16—C15—H15	118.4	C206—C205—C204	120.2 (2)
C14—C15—H15	118.4	C206—C205—H205	119.9
C15—C16—N24	125.3 (2)	C204—C205—H205	119.9
C15—C16—C17	123.8 (2)	C205—C206—C201	120.5 (2)
N24—C16—C17	110.6 (2)	C205—C206—H206	119.8
C18—C17—C16	107.3 (2)	C201—C206—H206	119.8
C18—C17—H17	126.4		
			
N22—Ni—N21—C1	−166.05 (18)	C14—C15—C16—N24	6.8 (4)
N24—Ni—N21—C1	13.61 (18)	C14—C15—C16—C17	−166.5 (2)
N22—Ni—N21—C4	20.04 (18)	C19—N24—C16—C15	−173.9 (2)
N24—Ni—N21—C4	−160.30 (18)	Ni—N24—C16—C15	6.0 (3)
N21—Ni—N22—C9	163.03 (18)	C19—N24—C16—C17	0.1 (2)
N23—Ni—N22—C9	−16.72 (18)	Ni—N24—C16—C17	−179.93 (15)
N21—Ni—N22—C6	−17.24 (18)	C15—C16—C17—C18	172.1 (2)
N23—Ni—N22—C6	163.01 (18)	N24—C16—C17—C18	−2.0 (3)
N22—Ni—N23—C14	−163.01 (18)	C16—C17—C18—C19	2.9 (3)
N24—Ni—N23—C14	17.33 (18)	C16—N24—C19—C20	−172.9 (2)
N22—Ni—N23—C11	11.83 (18)	Ni—N24—C19—C20	7.2 (3)
N24—Ni—N23—C11	−167.83 (18)	C16—N24—C19—C18	1.7 (2)
N21—Ni—N24—C16	165.54 (19)	Ni—N24—C19—C18	−178.24 (16)
N23—Ni—N24—C16	−14.71 (19)	C17—C18—C19—N24	−3.0 (3)
N21—Ni—N24—C19	−14.53 (19)	C17—C18—C19—C20	171.6 (2)
N23—Ni—N24—C19	165.21 (19)	N24—C19—C20—C1	6.7 (4)
C4—N21—C1—C20	169.3 (2)	C18—C19—C20—C1	−167.0 (2)
Ni—N21—C1—C20	−5.7 (3)	N24—C19—C20—C201	−179.9 (2)
C4—N21—C1—C2	−1.7 (2)	C18—C19—C20—C201	6.3 (4)
Ni—N21—C1—C2	−176.72 (15)	N21—C1—C20—C19	−7.6 (4)
N21—C1—C2—C3	2.7 (3)	C2—C1—C20—C19	162.5 (2)
C20—C1—C2—C3	−168.8 (2)	N21—C1—C20—C201	179.0 (2)
C1—C2—C3—C4	−2.4 (3)	C2—C1—C20—C201	−11.0 (3)
C1—N21—C4—C5	175.2 (2)	C58—O1—C51—O1A	49.5 (4)
Ni—N21—C4—C5	−9.9 (3)	C58—O1—C51—C5	165.0 (3)
C1—N21—C4—C3	0.2 (2)	C58—O1—C51—C52	−66.1 (4)
Ni—N21—C4—C3	175.18 (15)	C58A—O1A—C51—O1	−48.2 (4)
C2—C3—C4—N21	1.4 (3)	C58A—O1A—C51—C5	−163.5 (3)
C2—C3—C4—C5	−173.5 (2)	C58A—O1A—C51—C52	65.4 (4)
N21—C4—C5—C6	−11.2 (3)	C6—C5—C51—O1	−135.6 (3)
C3—C4—C5—C6	163.0 (2)	C4—C5—C51—O1	48.1 (3)
N21—C4—C5—C51	165.1 (2)	C6—C5—C51—O1A	−42.8 (4)
C3—C4—C5—C51	−20.7 (3)	C4—C5—C51—O1A	140.9 (3)
C9—N22—C6—C5	−176.1 (2)	C6—C5—C51—C52	92.2 (3)
Ni—N22—C6—C5	4.1 (3)	C4—C5—C51—C52	−84.1 (3)
C9—N22—C6—C7	−1.0 (2)	O1—C51—C52—C57	145.6 (3)
Ni—N22—C6—C7	179.22 (15)	O1A—C51—C52—C57	53.5 (4)
C4—C5—C6—N22	14.0 (3)	C5—C51—C52—C57	−81.5 (3)
C51—C5—C6—N22	−162.2 (2)	O1—C51—C52—C53	−38.2 (4)
C4—C5—C6—C7	−160.3 (2)	O1A—C51—C52—C53	−130.4 (3)
C51—C5—C6—C7	23.4 (3)	C5—C51—C52—C53	94.7 (3)
N22—C6—C7—C8	−1.6 (3)	C57—C52—C53—C54	0.6 (4)
C5—C6—C7—C8	173.5 (2)	C51—C52—C53—C54	−175.6 (2)
C6—C7—C8—C9	3.4 (3)	C52—C53—C54—C55	0.5 (4)
C6—N22—C9—C10	−168.4 (2)	C53—C54—C55—C56	−1.4 (4)
Ni—N22—C9—C10	11.4 (3)	C54—C55—C56—C57	1.1 (4)
C6—N22—C9—C8	3.1 (2)	C55—C56—C57—C52	0.0 (4)
Ni—N22—C9—C8	−177.11 (15)	C53—C52—C57—C56	−0.9 (4)
C7—C8—C9—N22	−4.2 (3)	C51—C52—C57—C56	175.4 (2)
C7—C8—C9—C10	167.4 (2)	C11—C10—C101—C102	109.2 (3)
N22—C9—C10—C11	5.4 (3)	C9—C10—C101—C102	−64.1 (3)
C8—C9—C10—C11	−164.9 (2)	C11—C10—C101—C106	−66.3 (3)
N22—C9—C10—C101	178.4 (2)	C9—C10—C101—C106	120.5 (2)
C8—C9—C10—C101	8.0 (3)	C106—C101—C102—C103	−0.8 (4)
C14—N23—C11—C10	174.9 (2)	C10—C101—C102—C103	−176.3 (2)
Ni—N23—C11—C10	−0.8 (3)	C101—C102—C103—C104	0.4 (4)
C14—N23—C11—C12	0.4 (2)	C102—C103—C104—C105	0.5 (4)
Ni—N23—C11—C12	−175.32 (15)	C103—C104—C105—C106	−1.0 (4)
C9—C10—C11—N23	−10.9 (3)	C104—C105—C106—C101	0.6 (4)
C101—C10—C11—N23	175.9 (2)	C102—C101—C106—C105	0.3 (4)
C9—C10—C11—C12	162.9 (2)	C10—C101—C106—C105	175.9 (2)
C101—C10—C11—C12	−10.3 (3)	C19—C20—C201—C202	116.7 (3)
N23—C11—C12—C13	0.9 (3)	C1—C20—C201—C202	−69.8 (3)
C10—C11—C12—C13	−173.7 (2)	C19—C20—C201—C206	−63.7 (3)
C11—C12—C13—C14	−1.8 (3)	C1—C20—C201—C206	109.9 (3)
C11—N23—C14—C15	172.7 (2)	C206—C201—C202—C203	1.5 (4)
Ni—N23—C14—C15	−11.5 (3)	C20—C201—C202—C203	−178.8 (2)
C11—N23—C14—C13	−1.5 (2)	C201—C202—C203—C204	0.4 (4)
Ni—N23—C14—C13	174.25 (14)	C202—C203—C204—C205	−1.7 (4)
C12—C13—C14—C15	−172.1 (2)	C203—C204—C205—C206	1.1 (4)
C12—C13—C14—N23	2.1 (3)	C204—C205—C206—C201	0.9 (4)
N23—C14—C15—C16	−4.0 (4)	C202—C201—C206—C205	−2.2 (4)
C13—C14—C15—C16	169.5 (2)	C20—C201—C206—C205	178.2 (2)
